# Editorial: Design and development of new therapeutics against infectious diseases using computational and experimental approaches

**DOI:** 10.3389/fcimb.2024.1453729

**Published:** 2024-07-05

**Authors:** Simone Brogi, Parth Sarthi Sen Gupta, Yogendra Kumar Mishra

**Affiliations:** ^1^ Department of Pharmacy, University of Pisa, Pisa, Italy; ^2^ School of Biosciences and Bioengineering, D. Y. Patil International University (DYPIU), Pune, Maharashtra, India; ^3^ Smart Materials, NanoSYD, Mads Clausen Institute, University of Southern Denmark, Sønderborg, Denmark

**Keywords:** infectious diseases, viruses, pathogens, bacteria, novel therapeutics

Because of growing concerns about future outbreaks caused by pathogens, we launched a Research Topic in 2022, namely “Design and Development of new Therapeutics Against Infectious Diseases Using Computational and Experimental Approaches” hosted by the journal *Frontiers in Cellular and Infection Microbiology*. In this scenario, the most important consideration should be the enrichment of current armamentariums in terms of antivirals (or broad-spectrum antivirals) and novel antibiotic agents considering that future pandemic conditions should interest emerging and re-emerging viruses with pandemic potential as well as increasing resistance to current antibacterial agents from emerging resistance-bacteria strains around the world, with particular interest in nosocomial infections and potentially untreatable disorders. Based on the World Health Organization (WHO), these events are expected to be future outbreaks that could damage public health (WHO-report-1, 2024; WHO-report-2, 2024). Due to this urgency, the integration of computational and experimental approaches can accelerate the development of novel strategies, thereby allowing the establishment of novel countermeasures against infectious diseases, thereby limiting disease progression and the possibility of increasing the number of potential events that could allow the expansion of these important threats. Accordingly, the combination of the mentioned approaches can significantly reduce, for example, the time required to identify promising compounds against infectious diseases, optimize their use, and possibly translate these compounds into clinical settings. On the other hand, there are different types of therapeutic agents that could be employed in the treatment of several infectious diseases, nevertheless, the potential resistance to many antibiotic agents and the insurgence of mutated viruses that can escape from vaccines; therefore, in these current cases, there is also a major need for the development of novel therapeutics to effectively treat infectious diseases. Moreover, relevance is assumed by the identification of novel delivery systems that improve the targeting of crucial pathogenic factors and the host immune response. Accordingly, with this aim, we introduce this Research Topic that attracted scientists in the field, and we were able to consider several submissions with a final number of published articles of 12. Among the articles of the Research Topic, 9 of them were Original Research articles, including 1 Case Report and 3 were Review articles, including 1 Mini-Review. These articles provided novel insights into the mechanisms of different infectious diseases and potential therapeutic approaches that could be useful for the development of novel antibacterial and antiviral agents.

Starting from research articles in the antiviral field, Xiong et al. applied a computational protocol based on network pharmacology, molecular docking, and molecular dynamics (MD) to explore the constituents of *Xuanfei Baidu* granule (XFBD) as anti-COVID-19 agents. Interestingly, the findings demonstrated that XFBD contains two significant active chemical components: I-SPD and chypodol. These components limit NLRP3 activation, which in turn reduces inflammatory response and apoptosis. By blocking the activation and chemotaxis of inflammatory cells via CSF2, I-SPD and vestitol can stop the progression of an inflammatory storm (Xiong et al.). Marriam et al. used different *in silico* procedures to identify a novel epitope-based peptide vaccine against the C30 endopeptidase regions of SARS-CoV-2. Furthermore, the proposed target was used in a virtual screening procedure to identify small molecules from the ZINC database that could interfere with endopeptidase function (Marriam et al.). Regarding the antiviral topic, a review by Sharma et al. was published under the Research Topic. In particular, they analyzed emerging evidence related to the Monkeypox infection. Among them, the global burden, resurgence, and possible management were reviewed in the mentioned paper (Sharma et al.).

Regarding the development of potential antimicrobial agents, some research articles were published under the Research Topic. Qasim et al. focused their work on the relevant topic of resistance pathogens. They conducted computer-aided genomic data analysis and bioinformatics techniques to identify potential new drug and vaccine targets against *Neisseria gonorrhoeae* infection. Interestingly, using a reverse vaccinology technique, two outer membrane proteins (AKP15153.1, AKP15828.1) were identified as potential vaccine candidates. Accordingly, a chimeric vaccine construct was generated using the top lead B- and T-cell overlapping epitopes. The molecular docking and MD simulation analyses indicated that the top-ranked vaccine candidate (V7) exhibited stable molecular interactions with human immune cell receptors (Qasim et al.). Schuurs et al. investigated covalent warheads and β-turn mimetic to identify LexA inhibitors coupling computational and experimental data. Two computational protocols, one for screening the β-turn mimetic and the other for considering a library of compounds containing covalent warheads that may target catalytic residue S119 to effectively inhibit the proteolytic function of LexA. Over 100 top-ranked molecules were tested using a RecA-mediated cleavage experiment to determine whether the compounds could prevent LexA cleavage in *E. coli*. Notably, a previously undiscovered covalent scaffold prevents RecA-mediated LexA cleavage was identified (Schuurs et al.). Shafaghi et al. conducted a study combining *in silico* and *in vitro* approaches to identify a novel candidate epitope-based vaccine targeting PspA PhtD of *Streptococcus pneumoniae*. Using immunoinformatics techniques, a fusion construct was created (PAD), by combining the immunodominant sections of PhtD (PD) with the immunodominant areas of PspA (PA) from families 1 and 2. This experiment aimed to evaluate the immunogenicity of the PAD fusion protein and assess its ability to protect against *S. pneumoniae* infection, both alone and in combination with PA and PD. Using computational techniques, the physicochemical properties, antigenicity, allergenicity, toxicity, and three-dimensional structure of the constructions were assessed. Molecular docking with the HLA receptor and immunological simulation were also performed. After immunizing mice, blood levels of antibodies and cytokines were measured, and the ability of antibodies to operate *in vitro* was assessed, as well as the survival rates of mice and the drop in bacterial loads in their blood and spleen. According to these findings, the fusion protein may be employed as a pneumococcal vaccine that is not dependent on serotype or as a useful ally protein in conjunction with a conjugate polysaccharide vaccine (Shafaghi et al.). Shen et al. described *in vitro* and *in vivo* experiments to determine the function of different *Lactobacillus* strains and their combinations in preventing *Helicobacter pylori* colonization and stomach mucosa irritation. Of all probiotics, *L. acidophilus* NCFM and *L. plantarum*, Lp-115 exerted a noteworthy effect on *H. pylori* elimination, reducing its adhesion and inhibiting the inflammatory response caused by *H. pylori* infection. The results of the present study provide significant value for the management of *H. pylori* infection, indicating the possibility of further clinical interventions with the two probiotic strains or their combination (Shen et al.). Wang et al. developed an *in silico* screening platform to identify possible chemicals able to activate AKT1 for the potential treatment of sepsis acute lung injury (SALI). Starting from the 3D structure of AKT1 (PDB ID 1UNR) and AKT activator molecules, the authors screened the ChemDiv database. Results showed that one of the top-ranked compounds (compound 7460-0250) selectively enhanced AKT1 phosphorylation and downregulated LPS-induced apoptosis in human umbilical vein endothelial cells (HUVECs) by activating the AKT-mTOR pathway. It was found that upregulated mTOR directly interacts with Bax to decrease apoptosis. *In vivo* studies have confirmed that the substance may lower SALI by activating the AKT-mTOR signaling pathway, reducing lung damage, and increasing the survival rate in mice with sepsis caused by cecum ligation and puncture (Wang et al.). An interesting meta-analysis of transcriptome from healthy and infected individuals, conducted by Ponnusamy and Arumugam, identified new potential therapeutic targets for drug repurposing studies by examining host directed drug-target interaction networks and protein interaction networks (human and *Mycobacterium tuberculosis*). Comparative research between healthy and tuberculosis cohorts provided insights into differentially expressed genes (DEGs) and made it possible to track these DEGs during vaccination or medication therapy. Furthermore, potential genes to target multidrug-resistant *M. tuberculosis* were identified. Among them, some kinases active in tuberculosis infection and ribosomal proteins, as well as proteins that enhance host-immune responses, were suggested as promising targets to be exploit for developing innovative anti-tubercular drug candidates (Ponnusamy and Arumugam). Again, a case report presented by You et al. described a patient with *Corynebacterium bovis* infection after fat breast augmentation. It is still unknown what caused the infection. The patient denied having been in an animal or tainted cow product, and she had no prior history of breast damage. Accordingly, this case report highlights the possible dangers of using fat-derived products that have been cryopreserved, including the risk of infection and adipocyte necrosis. The findings also emphasize the importance of comprehensive preoperative assessment and postoperative monitoring for the early detection and management of problems. Finally, high-throughput sequencing technology can be a useful diagnostic and therapeutic guide for diseases that do not respond to culture (You et al.). Finally, two review articles on the development of antimicrobial agents were published under the Research Topic. In the first article, Sahu et al. discussed the numerous coordinated efforts that have been made to date to create anti-*Candida* vaccines, along with a pan-fugal vaccine alternative. The authors have provided an updated context regarding vaccines undergoing clinical trials, obstacles, and potential future developments (Sahu et al.). Mayegowda et al. discussed nanoparticles (NPs) that can be employed for a variety of biological entities, including plants, bacteria, fungi, actinomycetes, viruses, and algae, each of which has a unique set of capabilities. Many pharmaceutical applications, including tissue engineering, pathogen or protein detection, antimicrobial agents, anticancer mediators, drug delivery vehicles, functional food formulations, and pathogen identification, can benefit from the use of NPs. These applications can also aid translational research into medical fields. In this review, the potential of eco-friendly synthesis to develop NPs as antimicrobial agents is described in detail (Mayegowda et al.).

In conclusion, we would like to express our gratitude to the Frontiers editorial staff for their generous ongoing support, as well as to all of the authors and co-authors for their significant contributions to this Research Topic and the reviewers for their invaluable effort in assessing the submitted manuscripts. We are also grateful to the guest editors. When all these efforts were combined, the Research Topic was successful. In addition to being a useful source of knowledge and inspiration for researchers and students, we anticipate that this topic will promote drug design and discovery for the treatment of infectious diseases. You can gain free access to the Research Topic by clicking on this link https://www.frontiersin.org/research-topics/37720/design-and-development-of-new-therapeutics-against-infectious-diseases-using-computational-and-experimental-approaches/magazine.

## Author contributions

SB: Conceptualization, Data curation, Formal analysis, Writing – original draft, Writing – review & editing. PS: Conceptualization, Data curation, Formal analysis, Writing – original draft, Writing – review & editing. YM: Conceptualization, Data curation, Formal analysis, Writing – original draft, Writing – review & editing.
